# Supplementary material for: A Humanized Yeast Phenomic Model of Deoxycytidine Kinase to Predict Genetic Buffering of Nucleoside Analog Cytotoxicity
Source: Genes (Basel). 2019 Sep 30;10(10):770. doi: 10.3390/genes10100770 (PMC6826991; doi:10.3390/genes10100770)
Supplement: Supplementary file 1 [file genes-10-00770-s001.zip › Additional_Files/Additional_File7_GOTermHeatmaps.zip!/Additional_File7_GOTermHeatmaps/ATP_synthesis_coupled_proton_transport.pdf]

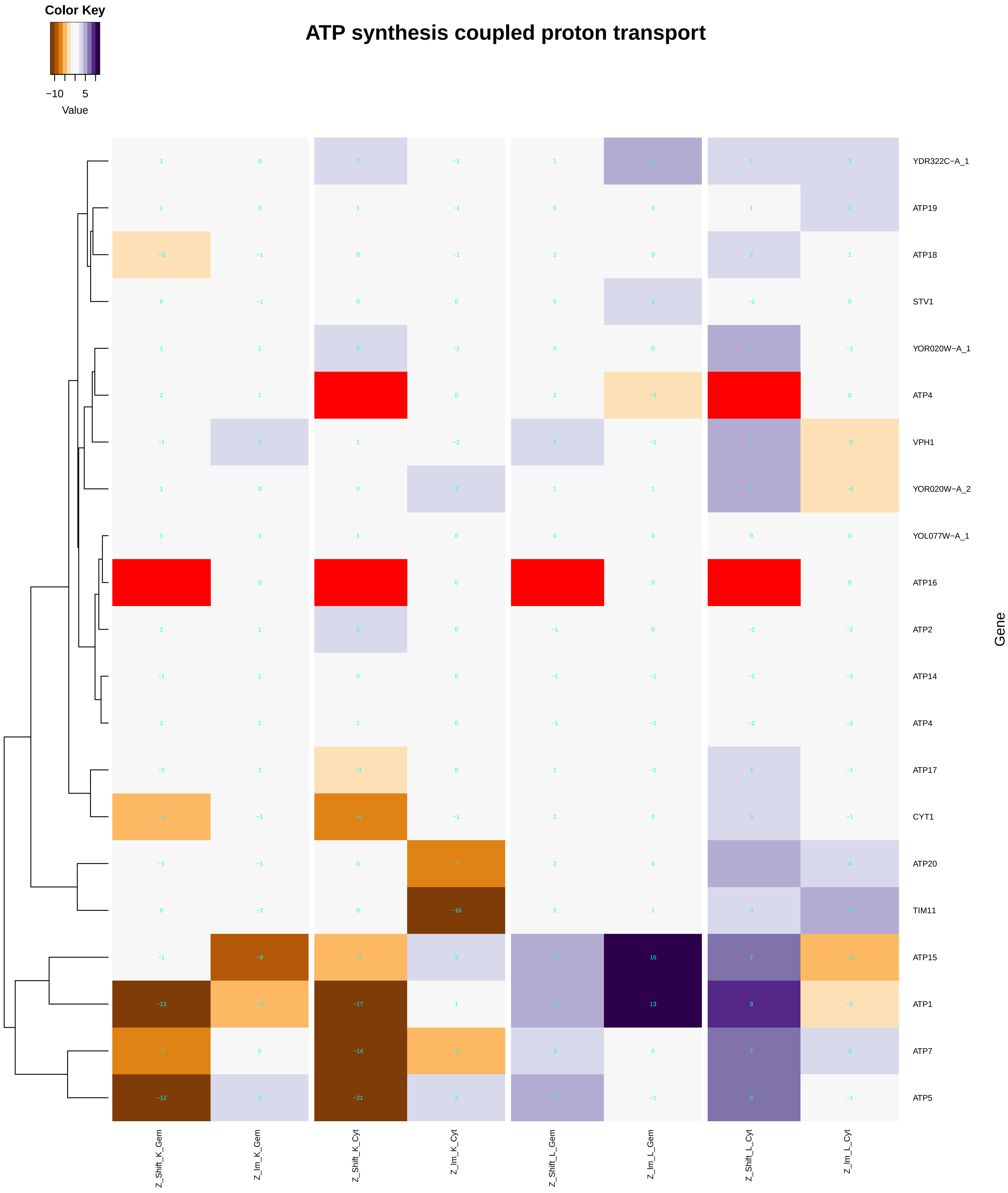
ATP synthesis coupled proton transport
Color Key
−10
5
Value
YDR322C−A_1
ATP19
ATP18
STV1
YOR020W−A_1
ATP4
VPH1
YOR020W−A_2
YOL077W−A_1
ATP16
ATP2
ATP14
ATP4
ATP17
CYT1
ATP20
TIM11
ATP15
ATP1
ATP7
ATP5
Gene
Z_Shift_K_Gem
Z_Im_K_Gem
Z_Shift_K_Cyt
Z_Im_K_Cyt
Z_Shift_L_Gem
Z_Im_L_Gem
Z_Shift_L_Cyt
Z_Im_L_Cyt
